# Antioxidant, antimicrobial and cytotoxicity potential of n-hexane extract of Cayratia trifolia L

**DOI:** 10.6026/97320630017452

**Published:** 2021-03-31

**Authors:** Bhuvaneswari Meganathan, Chella Perumal Palanisamy, Mani Panagal

**Affiliations:** 1 Department of Biotechnology, Annai College of Arts and Science (Affiliated to Bharathidasn University), Kovilacheri, Tamil Nadu 612503, India; 2 State Key Laboratory of Biobased Material and Green Papermaking, School of Food Science and Engineering, Qilu University of Technology, Shandong Academy of Science, Jinan 250353, China

**Keywords:** Cayratia trifolia, n-hexane extract, Antioxidant activity, Antimicrobial activity, Cytotoxicity activity

## Abstract

It is of interest to analyze the antioxidant, antimicrobial and cytotoxicity activity of n-hexane extract of Cayratia trifolia L. (C. trifolia). The antimicrobial activity of n-hexane extract of C. trifolia was determined using disc diffusion method against six selected pathogenic microorganisms. The cytotoxicity potential of n-hexane plant extract was also studied against A2780 cell lines by 3-(4,5-dimethylthiazol-2-yl)-2,5-diphenyl tetrazolium bromide (MTT) assay. Results, n-hexane extract of C. trifolia possess significant antioxidant activity with significant IC50 values in radical scavenging assays. In antimicrobial studies, the maximum zone of inhibition was found in the range of 19.0 ± 0.1 to 22.0 ± 0.1 mm. In MTT assay, inhibition of cell growth with minimal IC50 values of 46.25±0.42μg/mL against A2780 cell lines was observed. Thus, n-hexane extract of C. trifolia is a possible antioxidant, antimicrobial and cytotoxicity agent.

## Background

Multidrug resistance is one of the major health issues in worldwide and is further aggravated by overdose and misuse of existing antibiotics. In the recent years, more than 70% of the infections are due to the resistance to at least one of the antibiotics, which is mostly used for the treatment [1]. The progression of multidrug resistance to antibiotics and also the formation of reactive oxygen species (ROS) due to various intrinsic factors and extrinsic factors would lead to cause diseases and disorders including cardiovascular diseases, atherosclerosis, aging and inflammatory diseases. More recent studies have reported that, the excess generation of ROS might cause carcinogenesis. Therefore, globally cancer seems to be one of the foremost causes of morbidity and mortality, particularly in the developed countries [2]. Synthetic drugs, surgery, laser treatment, radiation therapy and chemotherapy are considered to be the general treatment methods for the treatment of cancer, but it might also exert toxicity to normal cells. The frequent use of synthetic drugs might lead to numerous side effects and occasionally drug resistance [3]. Unlike synthetic drugs, natural products possess significant importance in control and prevention of various disease and disorders without any serious side effects. Thus, the current scenario enforces the researchers to find out novel drug therapies especially from natural products. Natural and natural based products are always found to be effective in reducing the toxicity of allopathic drugs and therapy, therefore improving the survival rate of patients [4]. Medicinal plants contain different secondary metabolites including alkaloids, flavonoids, terpenoids and other phenolic compounds. These secondary metabolites possess strong antioxidant, cytotoxicity, antimicrobial, antidiuretic, antidiabetic, anti-inflammatory activities and also used to treat other disease and disorders, hence, play a major role in the management of human disease [5]. Hence, the present study was carried out in search of novel phytotherapeutic agents from the medicinal plant C. trifolia, which could be subjected to resolve this issue [6]. Cayratia trifolia L. (C. trifolia, Family: Vitaceae) commonly referred to as fox grape in English is native to India, Australia and few Asian countries [7]. Earlier preliminary phytochemical studies have confirmed the presence of yellow waxy oil, steroids, terpenoids, alkaloids, flavonoids such as kaempferol, myricetin, quercetin, triterpenes, epifriedelanol and tannins in the whole plant of C. trifolia [8-10]. The leaves of C. trifolia are rich in cyanidins and also found to contain piceid, resveratrol, viniferin, hydrocyanic acid, delphinidin and ampelopsin. The stem and roots of C. trifolia are also reported to possess hydrocyanic acid and delphinidin. Several studies on animal model has reported that C. trifolia possess antiviral, antibacterial, antiprotozoal, antitumor, cytotoxicity and antidiuretic properties. Traditionally, the decotion of seeds and tuber was given orally to diabetic patients to maintain the monitor the blood sugar level. The paste of the tuber is also used in the treatment of snake bite [11]. Therefore, the present study is aimed to validate the antioxidant, antimicrobial and cytotoxicity potential of n-hexane extract of whole plant of C. trifolia.

## Methodology

### Plant collection and authentication:

The whole plant of C. trifolia was collected from in the campus of Annai College of Arts and Science, Kovilacheri, Kumbakonam, Thanjavur District, Tamil Nadu, India and the plant was authenticated by Dr. P. Sathyanarayanan, Botanical survey of India, TNAU Campus, Coimbatore (voucher number is BSI/SRC/5/23/2010-2011/Tech.1527). The plant material was shade dried, powdered and stored in air tight container at 4°C for future analysis [12].

### Extract preparation:

The dried plant material was subjected to n-hexane extraction using exhaustive extraction procedure (Perumal et al., 2018b). Briefly, 200g of the plant material was soaked in a flask containing 1000 mL of n-hexane and was kept on the rotating shaker for 72 hours at 25°C (average room temperature). Finally, the collected extract was concentrated through rotary evaporator (RE-2A evaporator) set at 40°C. Further, it was stored at 4°C for future studies.

### Antioxidant assays:

The antioxidant potential of n-hexane extract of C. trifolia was determined through standard methods such as 1,1-diphenyl-2-picrylhydrazyl (DPPH) radical scavenging activity, hydroxyl radical scavenging activity, nitric oxide radical scavenging activity, reducing power assay and ferric reducing antioxidant potential (FRAP) assay.

### DPPH scavenging activity:

DPPH scavenging activity of n-hexane extract of C. trifolia was analysed according to Blois (1958) method. In brief, the 100 μM of DPPH was dissolved in ethanol, 1 mL of this mixture was added to 1 mL of different concentration (3.12-200 μg/ml) of the plant extract, mixed vigorously and allowed to stand at room temperature for 30 minutes. The decrease in absorbance values were observed at 517 nm against blank solution (ethanol). DPPH scavenging capacity was calculated by reduction of radical percentage. Each test was analysed in triplicate. The decreased absorbance values signified higher free radical scavenging activity.

### Nitric oxide scavenging activity:

Nitric oxide was produced through sodium nitroprusside (SNP) and it was analysed by Garratt (1964) method. Briefly, the effective combination contains 10 mM of SNP, phosphate buffer (pH 7.4) and different concentrations (3.12-200 μg/ml) of the extract in the total volume of 3 mL. It was incubated at room temperature for 150 minutes, then 1 mL of sulfanilamide was mixed in 0.5 mL of the incubated solution and was again allowed to stand at room temperature for 5 minutes. Finally, 1 mL of napthylethylenediamine dihydrochloride (NED) (0.1% w/v) was added and the mixture was incubated for another 30 minutes at room temperature. The pink chromophore was produced during the diazotization of nitrite ions with sulphanilamide and subsequent coupling with NED was analysed spectrophotometrically at 540 nm against the blank solution. Each test was performed in triplicate.

### Hydroxyl scavenging activity:

Hydroxyl radical scavenging activity of n-hexane extract was analysed by the proposed method of Elizabeth and Rao (1990) [13] with a slight modification. Freshly prepared 1 mL of the reaction mixture [2-deoxy-2-ribose (2.8 mM), KH2PO4-KOH buffer (20 mM, pH 7.4), FeCl3 (100 μM), EDTA (100 μM), H2O2 (1.0 mM), ascorbic acid (100 μM)] and different concentrations (3.12-200 μg/ml) of n-hexane extract was incubated at 37°C for 1 hour, followed by the addition of 0.5 ml of the reaction mixture and 1 ml of 2.8% TCA and 1% aqueous TBA. Again the contents in the test tubes was incubated at 90°C for 15 minutes (for the colour development). The absorbance was calculated at 532 nm against a suitable blank solution. Each test was analysed in triplicate.

### Reducing power assay:

The reducing power assay of n-hexane extract was measured by the method proposed by Oyaizu (1986) [14]. In brief, the different concentrations (3.12-200 ug/mL) of the plant extract (0.5 mL) were added with 0.5 mL phosphate buffer (0.2 M, pH 6.6) and 0.5 ml potassium hexacyanoferrate (0.1%), then it was incubated at 50°C in the water bath for 20 minutes. Later, 0.5 ml of 10% TCA was added to the mixture. 1 mL of the above solution was taken followed by the addition of 1 ml of distilled water and 0.1 mL of 0.01% FeCl3 solution. Then the mixture was allowed to stand for 10 minutes at 25°C. The absorbance was calculated at 700 nm against the appropriate blank solution. Each test was analysed in triplicate. The higher absorbance values signify its greater reducing power.

### FRAP reducing assay:

The antioxidant capacity of n-hexane extract was measured using FRAP assay proposed by Chakraborthy et al., (2010) [15]. The conversion of ferric ion (Fe3+) to ferrous ion (Fe2+) finally to produce a blue complex of Fe2+/2,4,6-Tri(2-pyridyl)-s-triazine (TPTZ) was observed , which enhanced the absorption at 593 nm. In brief, FRAP reagent was prepared by the mixture of solution contains 10 μM of TPTZ, 40 μM of HCl, 20 μM of FeCl3 at 10:1:1 ratio and 0.3M acetate buffer. It was prepared freshly and allowed to stands at 37°C. 800 μL of the reagent was mixed with 80 μL of sample solution that contains various concentrations of the plant extract (3.12-200 μg/mL). This mixture was vigorously shaken and allowed to stand at 37°C for 30 minutes. The absorbance was measured at 595 nm. All the analysis was done in triplicate.

### Antimicrobial activity:

The antimicrobial activity of n-hexane extract was determined through Bauer et al (1966) [16] proposed method. In short, the procedure is described as follows. The sterile disc (5 mm) was dipped in various concentrations of the plant extract (5, 10 and 15 mg/ml) and dried at 40°C. Each disc drenched in dimethyl sulfoxide (DMSO) and chloromophenicol was used for negative and positive control respectively. The inoculum [108 CFU] was broaden on the sterile of nutrient agar medium plate using cotton swabs and the plates were incubated at 35°C for 20 minutes. The discs were placed on the plates and was incubated at 37°C for 24 hours. Correspondingly, antifungal activity was measured by the afore mentioned method in Sabouraud Dextrose Agar (SDA) medium. The standard streptomycin (10μg/disc) was used as positive control. Each plate was incubated at 25°C for 72 hours. The zone of inhibition was calculated in mm. Each test was performed in triplicate.

### Cytotoxicity analysis:

The cytotoxicity assay was determined by MTT assay [17]. Briefly, 5000 cells were seeded in each well on 96 well plates and cultured for 24 hours, then treated with different concentration (3.12, 6.25, 12.5, 25, 50, 100, 200 μg/mL) of plant extract while cyclophosphamide was used as positive control. The cells were then incubated at 37°C for 24 hours in 5% CO2. At the end of the incubation, the medium was removed and 10 μL of MTT was added followed by 100 μL of DMSO was added to each well to solubilize the formazan crystals. It was then left in dark at room temprature. The absorbance was measured at the wavelength of 595 nm using a mircotitre plate reader and the results were analysed in triplicate and the percentage was calculated.

### Statistical analysis:

The obtained results from the assays were showed as mean ± SD. The Statistical evaluations were measured through statistical package program (SPSS 10.0, IBM, Armonk, New York, United States).

## Results and Discussion:

**Figure 1:**
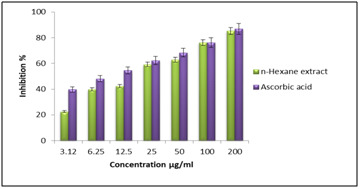
DPPH radical scavenging activity of n-hexane extract.

**Figure 2:**
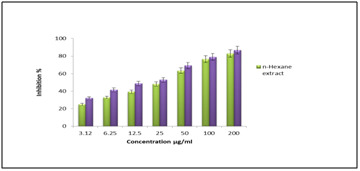
Nitric oxide radical scavenging activity of n-hexane extract.

**Figure 3:**
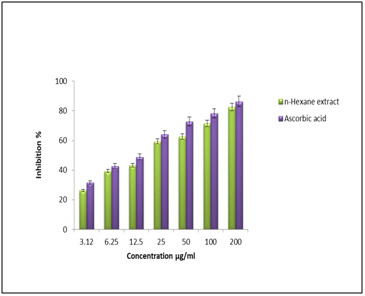
Hydroxyl radical scavenging activity of n-hexane extract.

**Figure 4:**
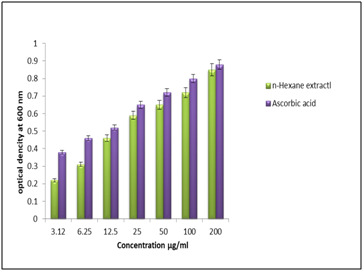
Reducing power activity of plant extract.

**Figure 5:**
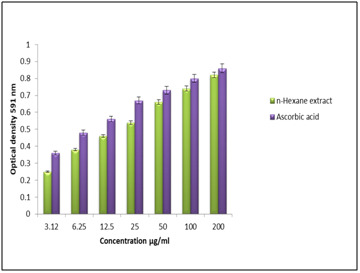
FRAB assay activity of plant extract.

**Figure 6:**
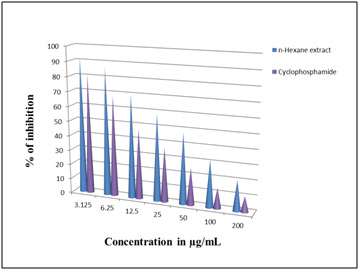
Cell growth inhibitory assay.

**Figure 7:**
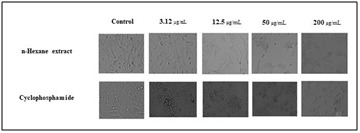
A2780 cell lines treated with n-hexane extract and standard drug.

Antioxidants are widely utilized as food additives in industries to avoid food degradation due to the production of free radicals, which in turn might lead to progression of ailments and illness in humans [18]. Novel methods are proposed to measure the antioxidant activity of phytocompounds but the standard scavenging assays followed in this study is based on their free radical scavenging capacities [19]. It is a well-known procedure to determine the antioxidative properties of phytocompounds [20]. The free radicals produced are scavenged by an antioxidant which donates an electron or hydrogen ion to a radical and consequently, a constant molecule is produced [21]. The dose-dependent DPPH scavenging effect of n-hexane extract expressed the comparable and significant IC50 values of 19.86±0.21 μg/mL when compared with ascorbic acid value of 09.52±0.13 μg/mL (Figure 1 and Table 1). Nitric oxide is a well-known pro-inflammatory mediator which is concerned in variety of physiological events like, smooth muscle relaxant, inhibition of platelet aggregation and regulation of cell mediated toxicity [22]. However, over production of nitric oxide may lead to pathogenesis of inflammatory diseases. Therefore, nitric oxide inhibitory agent could be favorable for the management of inflammatory reactions [23]. The nitric oxide scavenging activity of n-hexane extract holds the significant IC50 value of 28.64±0.16 μg/mL (Figure 2 and Table 1) when compared with the standard ascorbic acid standard (22.59±0.09 μg/mL). The results indicate that n-hexane extract of C. trifolia possess nitric oxide scavenging activity.The production of hydroxyl radicals by Fenton reaction can degrade deoxy ribose and cause the oxidative DNA damage. In this study, n-hexane extract and reference drug of ascorbic acid exposed their significant IC50 values were 20.39±0.12 μg/mL and 14.56±0.07 μg/mL respectively (Figure 3 and Table 1). One of the major ROS, hydroxyl radicals can increase the progression lipid peroxidation and in turn could cause overall biological damage in the body [24]. The results obtained also suggest that n-hexane extract of C. trifolia might be capable of eliminating malondialdehyde, the most mutagenic product of lipid peroxidation. Mostly, it can be used to assess the capability of an antioxidant as an electron donor [25]. In this assay, the reducing power capacity of n-hexane extract and reference drug ascorbic acid was analyzed through the transformation of Fe3+ to Fe2. At the concentration of 200 μg/ml, the highest absorbance of n-hexane extract and reference drug was found to be 0.85±0.02 and 0.88±0.01 respectively (Figure 4). This result indicates that n-hexane extract of C. trifolia has significant reducing power activity. Hence, it could scavenge free radicals by donating an electron, which might terminate the progression of lipid peroxidation chain reaction. This assay is mainly based on the decreasing effect of an antioxidant responding to a ferric tripyridyltriazine complex and generating the colored ferrous tripyridyltriazine. The reducing power of n-hexane extract of C.trifolia could be due to hydrogen atom donation. This process might terminate the free radical chain reaction [26]. The n-hexane extract of C. trifolia showed highest absorbance of 0.82±0.03, whereas ascorbic acid showed 0.86±0.02 at the concentration of 200 μg/ml (Figure 5) This confirms the significant reducing power activity of n-hexane extract of C. trifolia. Thus, the antioxidant activity of n-hexane extract of C. trifolia exposed the dose dependant percentage of scavenging activities which is expressed as half maximal inhibitory concentration (IC50) value. The antimicrobial activity of C. trifolia extract and reference drugs was studied by disc diffusion method (Table 2) against six selected microorganisms, which are regularly involved in the development of infectious diseases. The antimicrobial activity of the extract expressed the highest inhibition zone against the tested microorganisms such as, Staphylococcus aureus (19.0 ± 0.4 mm), Streptococcus aureus (19.0 ± 0.2 mm), Escherichia coli (22.0 ± 0.1 mm), Klebsiella pneumoniae (20.0 ± 0.3 mm), Aspergillus niger (21 .0± 0.2 mm) and Aspergillus terreus (19.0 ± 0.1 mm). Multidrug resistance is growing rapidly during recent decades. Recent research studies have urged the need to search new drug sources of improved therapeutic properties [27]. In the present study, the results indicated that the n-hexane extract of C. trifolia have significant antimicrobial activity against the disease causing microorganisms and it could have the potential bactericidal effects on those microorganisms. The in vitro cytotoxicity activity of n-hexane extract of C. trifolia was investigated using different concentrations ranging from 3.12 to 200 μg/mL against A2780 ovarian cancer cell lines. N-hexane extract showed 86% of cell growth inhibition (Figure 6) at the highest concentration of 200 μg/mL, where as cyclophosphamide showed 88% ie, significant cell growth inhibitory activity (IC 50 value) by n-hexane extract (Figure 7) was observed to be 46.25±0.42μg/mL, when compared to the standard, cyclophosphamide. The present study has confirmed that the induction of cell death occured at a very low concentration like any other potential cytotoxicity drug [28].Thus, it may be considered to be a good candidate for therapeutic agent.

## Conclusion

The n-hexane extract of C. trifolia showed free scavenging activity of DPPH. The antimicrobial activity of n-hexane extract of C. trifolia with six selected microorganisms is shown. The extract showed strong cell growth inhibitory activity on A2780 ovarian cancer cell lines. Thus, n-hexane extract of C. trifolia showed antioxidant, antimicrobial and Cytotoxicity activity.

## Figures and Tables

**Table 1:** Antioxidant potential of n-hexane extract

Extracts	Inhibitory concentration (IC50 values) in μg/ml		
	DPPH scavenging activity	Nitric oxide scavenging activity	Hydroxyl scavenging activity
n-Hexane extract	19.86±0.21	28.64±0.16	20.39±0.12
Ascorbic acid	09.52±0.13	22.59±0.09	14.56±0.07

**Table 2:** Antimicrobial capacity of n-hexane extract

No.	Microorganism species	Inhibitory zone at 15 μg/mL	
		n-Hexane extract (mm)	Antibiotic (mm)
1	Staphylococcus aureus	19.0 ± 0.4	22.0 ± 0.1
2	Streptococcus aureus	19.0 ± 0.2	21.0 ± 0.
3	Escherichia coli	22.0 ± 0.1	21.0 ± 0.2
4	Klebsiella pneumoniae	20.0 ± 0.3	22.0 ± 0.2
5	Aspergillus niger	21 .0± 0.2	23.0 ± 0.3
6	Aspergillus terreus	19.0 ± 0.1	22.0 ± 0.1
